# Molecular Basis for the Regulation of Transcriptional Coactivator p300 in Myogenic Differentiation

**DOI:** 10.1038/srep13727

**Published:** 2015-09-10

**Authors:** Jihong Chen, Yingjian Wang, Munerah Hamed, Natascha Lacroix, Qiao Li

**Affiliations:** 1 Department of Cellular and Molecular Medicine, Faculty of Medicine, University of Ottawa, Ottawa, Ontario, Canada; 2 Department of Pathology and Laboratory Medicine, Faculty of Medicine, University of Ottawa, Ottawa, Ontario, Canada

## Abstract

Skeletal myogenesis is a highly ordered process which specifically depends on the function of transcriptional coactivator p300. Previous studies have established that Akt/protein kinase B (PKB), a positive regulator of p300 in proliferating cells, is also important for proper skeletal muscle development. Nevertheless, it is not clear as to how the p300 is regulated by myogenic signaling events given that both p300 and Akt are involved in many cellular processes. Our studies revealed that the levels of p300 protein are temporally maintained in ligand-enhanced skeletal myocyte development. Interestingly, this maintenance of p300 protein is observed at the stage of myoblast differentiation, which coincides with an increase in Akt phosphorylation. Moreover, regulation of p300 during myoblast differentiation appears to be mediated by Akt signaling. Blunting of p300 impairs myogenic expression and myoblast differentiation. Thus, our data suggests a particular role for Akt in myoblast differentiation through interaction with p300. Our studies also establish the potential of exploiting p300 regulation and Akt activation to decipher the complex signaling cascades involved in skeletal muscle development.

Many diseases and conditions including aging, cancer, AIDS, congestive heart failure and chronic obstructive pulmonary diseases, can lead to muscle wasting disorders that are extremely debilitating^1^. Although stem cell-based therapies present great promise to prevent or reverse the lasting effects of muscle atrophy, many challenges remain. Understanding the molecular basis of myogenic differentiation is thus a critical step in developing the best strategy to direct stem cell-based muscle regeneration.

The development of myoblasts from myogenic progenitors, and subsequent cell cycle arrest and differentiation into mature skeletal muscle, are a highly ordered processes controlled by multiple myogenic regulatory factors, including Myf5, MyoD and myogenin^2^,^3^. While Myf5 and MyoD initiate the commitment of skeletal muscle lineage and formation of myoblasts, terminal differentiation and fusion of myoblasts into myotubes is governed by myogenin^4^. In addition, genetic evidence from the mouse and ES cell model systems has established that the histone acetyltransferase (HAT) activity of p300 is essential for the expression of Myf5 and MyoD, and consequently for skeletal muscle development^5^.

Initially identified as an E1A-associated protein^6^,^7^, p300 is an important regulator of cell function through its intrinsic HAT activity and its capacity to interact with different transcription factors and coactivators^6^,^7^,^8^,^9^,^10^. As a result, p300 occupancy is the best chromatin signature of enhancers^11^,^12^,^13^. Embryonic development is very sensitive to p300 gene dosage, and the p300 null cells are particularly defective in retinoid acid (RA) signaling^14^. While p300 can be a substrate for phosphorylation, ubiquitination and acetylation^15^,^16^,^17^,^18^,^19^,^20^,^21^, less is known on how p300 function is specifically regulated in response to the developmental cue of skeletal myogenesis.

Akt/protein kinase B (PKB) is a serine/threonine kinase that is important for signaling in many cellular processes including p300 phosphorylation and regulation^21^,^22^,^23^. There are three isoforms of Akt (Akt1, Akt2 and Akt3) in mammals. Phosphorylation of the conserved serine and threonine residues is necessary for Akt enzymatic activity in all three isoforms^24^. While Akt1 is the predominant isoform expressed in most tissues, Akt2 is highly expressed in skeletal muscle, the heart, liver and kidneys^25^. The expression of Akt3 is more limited and found mostly in the testes and brain^26^. Thus, most studies focus on Akt1, often referred simply as the Akt in the literature. Nevertheless, it is increasingly evident that Akt1 is mainly involved in cellular survival pathways and Akt2 in glucose homeostasis, whereas the function of Akt3 is less clear but has been linked to brain development^27^,^28^,^29^,^30^,^31^,^32^.

P19 pluripotent stem cells have been used extensively to study the molecular mechanism of stem cell differentiation^33^. They form embryo bodies (EBs) readily and respond to various treatment conditions to undergo lineage-specific differentiation^34^. For example, treatment of the EBs with 1% of DMSO induces the development of a small percentage of skeletal myocytes, while the addition of RA significantly enhances the expression of Pax3 and Myf5 (Fig. 1), hence increases the efficacy of skeletal muscle development^35^,^36^. On the other hand, C2C12 is a non-transformed myogenic cell line obtained by continuous passaging of primary myoblasts isolated from mouse limb muscle^37^. These cells are well characterized and closely resemble proliferating myoblasts that express the Myf5 and MyoD determination factors. As such, they proliferate as committed myoblasts when cultured with growth factors, but differentiate in low mitogen conditions and undergo terminal differentiation and fusion to form multi-nucleated myotubes (Fig. 1). The C2C12 cells are also amenable to genetic manipulation to incorporate and express ectopic genes allowing the selection of stable clones that retain the capacity to differentiate.

Intriguingly, while the p300 appears to be ubiquitously involved in a myriad of cellular processes, its HAT activity is specifically required for skeletal myogenesis *in vivo*^5^. Thus, it is imperative to comprehend on a molecular level how different signaling pathways cross talk to regulate p300 function during skeletal muscle development. In this study, we have examined the impact of signaling-dependent events on p300 and myogenic differentiation. In this regard, our studies reveal a role for lineage specific signaling and p300 regulation, and implicate a specific Akt activity in this process.

## Results

### Efficacies of RA on myogenic conversion

It is well established that during pluripotent stem cell differentiation, RA affects the efficacy and pattern of stem cell differentiation in a concentration-dependent manner^38^,^39^. Consistent with previous reports, we observed that at a low concentration (10 nM), RA in the presence of DMSO significantly enhanced the differentiation of P19 stem cells into skeletal myocytes that also exhibited a more intensive staining of the myosin heavy chain (Fig. 2A,B). However, at a higher concentration (1 μM), RA completely blocked the myogenic conversion (Fig. 2A,B).

More interestingly, we also observed a significant fluctuation in p300 protein levels during the myogenic conversion. Following DMSO treatment, the level of p300 protein decreased by about 85% on day 9 of differentiation, in comparison to day 4 (Fig. 2C,D). The addition of RA at 10 nM which is an optimal condition for myogenic conversion, significantly halted the reduction of p300 on day 9, as about 85% of p300 protein remained in comparison to day 4 (Fig. 2A–E). In contrast, the addition of RA at 1 μM that blocks the development of skeletal myocytes had no such effect (Fig. 2A–E). Moreover, Western blotting demonstrated that myogenin protein, a muscle specific factor, was readily detected following the addition of 10 nM RA, but not the high concentration of 1 μM (Fig. 2F). Thus, this maintenance of p300 protein subsequent to RA treatment appears to be an integral part of skeletal muscle development.

### Akt activity in myogenic conversion

Since Akt is a positive p300 regulator in proliferating cells^23^, we next examined the link between Akt and p300 in myogenic conversion. As shown in Fig. 3A,B, the maintenance of p300 protein following RA and DMSO treatment during the P19 myogenic conversion coincided with a significant increase of Akt phosphorylation at Ser473, while the relative abundance of total Akt protein and mRNA did not change that much (Fig. 3A,C).

To probe for the contribution of Akt to RA-enhanced myogenic conversion, we took an inhibitor approach by using Akt inhibitor IV, a pan-Akt inhibitor^40^. Since the maintenance of p300 is observed subsequent to RA treatment, post EB formation, i.e., at the stages of myoblast differentiation, we treated the pluripotent stem cells with the Akt inhibitor after EB formation, from day 5 to 8 of differentiation (Fig. 3D, bottom panel). As shown Fig. 3D, this Akt inhibitor significantly impaired RA-enhanced skeletal myocyte development. Taken together, our data suggests that the function of Akt isoforms may be important for a p300-dependent myoblast differentiation.

### Akt activity in myoblast differentiation

**Figure 1:**
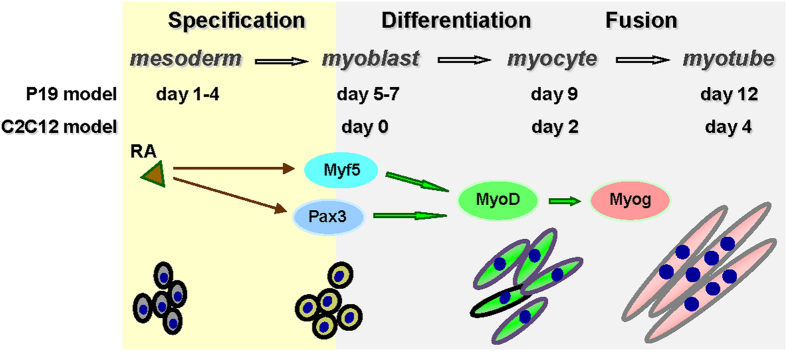
Schematic presentation of cell model systems used for myogenic differentiation. RA together with DMSO enhances the commitment of skeletal muscle lineage through the regulation of Pax3 and Myf5 gene expression in pluripotent stem cells (day 1–4, solid brown arrows). Pathways required for consequent myoblast differentiation and fusion events are denoted with open green arrows.

**Figure 2:**
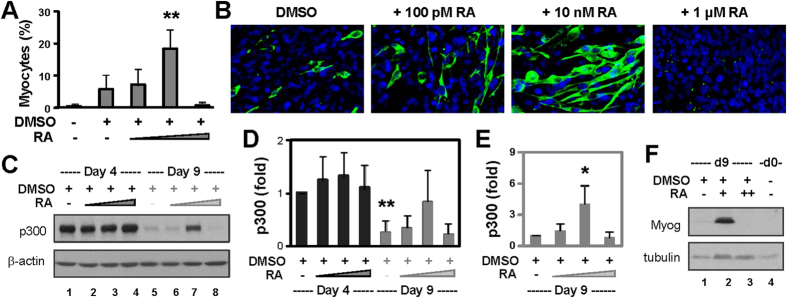
Effects of RA on p300 and myogenic conversion during stem cell differentiation. (**A**) P19 pluripotent stem cells were treated with increasing concentrations of RA (100 pM, 10 nM, 1 μM) and DMSO during EB formation and cultured for additional 5 days without any treatment. The cells were stained for myosin heavy chain for quantitative microscopy. Efficacies of myogenic differentiation are presented as the percentage of skeletal myocytes in relation to the total cell populations. Error bars are the standard deviations of three independent experiments (**p < 0.01 relative to DMSO control). (**B**) Shown are the representative images of myosin heavy chain (green) and nuclei (blue) co-staining on day 9 of differentiation. (**C**) The levels of p300 protein on day 4 and 9 of differentiation were examined by Western. The blots were stripped and reprobed for β-actin as loading controls. Shown are the cropped blot images representing indicated proteins. (**D**) Quantification of the blots is presented as the fold change of p300 in relation to day 4 DMSO control normalized to β-actin (**p < 0.01, n = 4). (**E**) Levels of p300 on day 9 are also plotted as the fold change in relation to the day 9 DMSO control (*p < 0.05). (**F**) Myogenin expression was examined by Western blotting. The blots were then stripped and reprobed for β-tubulin. Shown are the cropped blot images representing indicated proteins.

**Figure 3:**
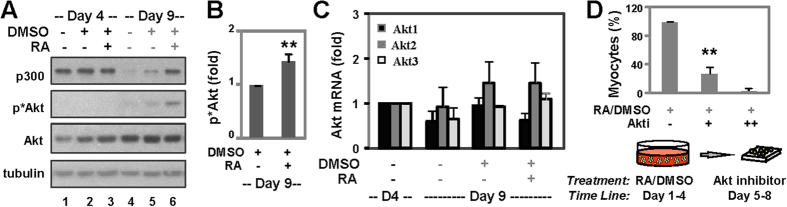
Akt activity during myogenic conversion. (**A**) P19 cells were treated with RA (10 nM) and DMSO during EB formation and cultured for additional 5 days without treatments. Levels of p300, Akt and phosphorylated Akt were analyzed by Western blotting. The blots were then stripped and reprobed for β-tubulin as loading controls. Shown are the cropped blot images representing indicated proteins. (**B**) Quantification of the phosphorylated Akt blots is presented as the fold change of day 9 DMSO control normalized to β-tubulin (*p < 0.05, n = 5). (**C**) The mRNA levels of Akt1, Akt2, and Akt3 were examined by qPCR. Quantification is presented as the fold change relative to respective isoforms in untreated day 4 controls with GAPDH as an internal control (n = 3). (**D**) Following EB formation, the cells were treated with Akt inhibitor IV (0.5, 1.0 μM), and stained for myosin heavy chain on day 9 of differentiation. Results from the quantitative microscopy are presented as the percentage of skeletal myocytes relative to control without inhibitor treatment (**p < 0.01, n = 3).

To study the interplay of p300 and Akt activity in myoblast differentiation, we next employed the C2C12 model in our study as the early stages of C2C12 differentiation (day 1–2) correlate well with the late stages of P19 myogenic conversion (day 5–9, Fig. 1), wherein the abundance of p300 protein and phosphorylated Akt positively correlate with the efficacies of myogenic conversion (Figs 2 and 3). In addition, it has been reported that Akt is activated and stabilized during C2C12 differentiation^41^.

**Figure 4:**
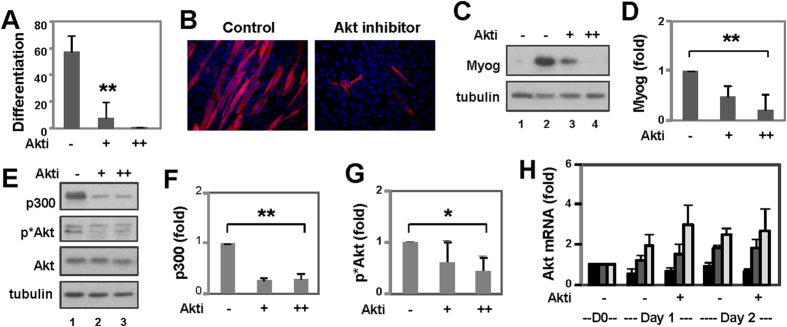
Effects of an Akt inhibitor on p300 and C2C12 myoblast differentiation. (**A**) C2C12 myoblasts were differentiated for 1–3 days in the presence of Akt inhibitor IV (0.5, 1.0 μM) and stained on day 3 for quantitative microscopy. Differentiation was defined as the percentage of myocyte nuclei relative to the total number of nuclei. Error bars are the standard deviations of five independent experiments (**p < 0.01). (**B**) Shown are the representative images of myosin heavy chain (red) and nuclei (blue) co-stain. (**C**) The levels of myogenin protein were examined by Western on day 2 of differentiation. The blots were then stripped and reprobed for β-tubulin as loading controls. Proliferating cells (D0) were also included as control. Shown are the cropped blot images representing indicated protein. (**D**) Quantification of the myogenin blots is presented as the fold change of untreated differentiating myoblasts normalized to β-tubulin (**p < 0.01, n = 4). (**E**) Levels of p300, Akt and phosphorylated Akt were analyzed by Western on day1 of differentiation. The blots were then stripped and reprobed for β-tubulin. Shown are the cropped blot images representing indicated proteins. Full-length blots are presented in the Supplementary Figure S1. (**F**) Quantification of the p300 blots is presented as the fold change of untreated differentiating myoblasts normalized to β-tubulin (**p < 0.01, n = 4). (**G**) Quantification of the phosphorylated Akt blots is presented as the fold change relative to untreated differentiating myoblasts (*p < 0.05, n = 4). (**H**) qPCR analysis of the mRNA levels of Akt1, Akt2, and Akt 3 is presented as the fold change relative to its respective isoform in proliferating cells (D0) using GAPDH as internal controls.

The C2C12 cells were differentiated in the presence of Akt inhibitor IV for 1–3 days. Quantitative microscopy demonstrated that similar to the P19 myogenic conversion, the pan Akt inhibitor significantly inhibited the formation of skeletal myocytes from the C2C12 myoblasts (Fig. 4A,B). This inhibitory effect of Akt inhibitor on myoblast differentiation was also corroborated by a significant inhibition of myogenin expression as determined by Western blotting (Fig. 4C,D).

Moreover, treatment of the C2C12 cells with Akt inhibitor IV significantly decreased the levels of p300 protein, while inhibiting myoblast differentiation (Fig. 4E,F). Western blotting also revealed that the steady-state levels of total Akt protein were not much affected by the Akt inhibitor (Fig. 4E). However, the levels of Akt phosphorylation at Ser473 decreased significantly following treatment with the Akt inhibitor (Fig. 4E,G).

Interestingly, the mRNA levels of Akt1 decreased moderately during myoblast differentiation, while that of Akt2 and Akt3 showed some increase (Fig. 4H). More importantly, treatment with Akt inhibitor IV did not negatively affect the mRNA levels of Akt isoforms during myoblast differentiation (Fig. 4H). Taken together, our data indicates that during C2C12 differentiation, Akt inhibitor IV indeed acts through Akt impairment as this inhibitor targets the ATP-binding site of a kinase upstream of Akt, and thus inhibits the phosphorylation/activation of Akt^40^.

### Role of p300 in myoblast differentiation

**Figure 5:**
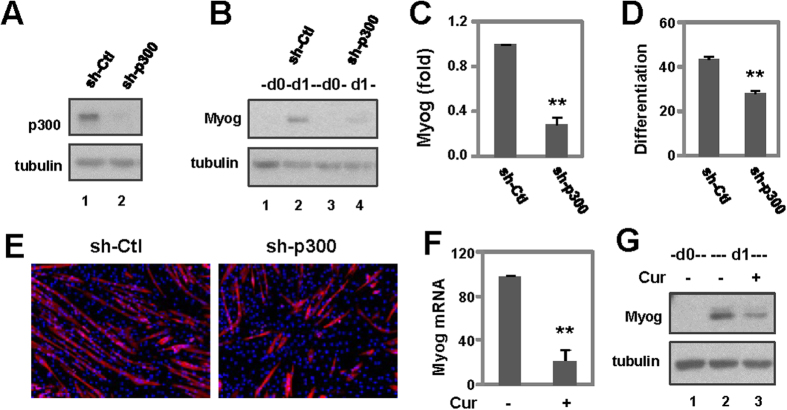
Role of p300 in myoblast differentiation. (**A**) Levels of p300 protein were analyzed using Western blotting following the introduction of p300 shRNA (sh-p300) into the parental C2C12 cells. The blots were then stripped and reprobed for β-tubulin as loading controls. Shown are the cropped blot images representing indicated proteins. A nonsilencing shRNA (sh-Ctl) was used as a negative control. (**B**) The levels of myogenin protein were analyzed on day1 of differentiation using Western blotting. The blots were then stripped and reprobed for β-tubulin. Proliferating cells and cells infected with the nonsilencing shRNA were used as controls. Shown are the cropped blot images representing indicated proteins. (**C**) Quantification of the myogenin blots is presented as the fold change relative to the nonsilencing shRNA control normalized to β-tubulin (**p < 0.01, n = 4). (**D**) The cells were differentiated for 4 days and then stained for quantitative microscopy. Differentiation was defined as the percentage of myocyte nuclei relative to the total number of nuclei. Error bars are the standard deviations of four independent experiments (**p < 0.01). (**E**) Shown are the representative microscopic images of myosin heavy chain (red) and nuclei (blue) co-stain. (**F**) The C2C12 cells were differentiated in the presence of curcumin (Cur, 10 μM), and subjected for qPCR analysis of myogenin mRNA on day 1 of differentiation. Quantification is presented as the fold change relative to untreated control using GAPDH as internal controls (**p < 0.01, n = 3). (**G**) Myogenin protein was examined by Western analysis in parallel. The blots were then stripped and reprobed for β-tubulin as loading controls. Shown are the cropped blot images representing indicated proteins.

To further determine the role of p300 in myoblast differentiation, we used a shRNA approach to knockdown the endogenous p300 protein for a loss-of-function study. Western blotting showed that introduction of the p300 shRNA into the proliferating myoblasts effectively knocked down the endogenous p300 protein (Fig. 5A). However, infection of a non-silencing control shRNA did not affect the level of endogenous p300 (Fig. 5A). Next, we examined the effect of p300 knockdown on myogenic expression. As shown in Fig. 5B,C, introduction of the p300 shRNA significantly decreased the expression of myogenin protein by about 75%. Moreover, the development of skeletal myocytes was significantly inhibited following p300 knockdown as determined by quantitative microscopic analysis (Fig. 5D,E). Thus the function of p300 is essential for myoblast differentiation.

To examine the role p300 HAT activity in myogenic expression, we also employed curcumin as it inhibits the HAT activity of p300 and consequently myoblast differentiation^42^,^43^. The C2C12 cells were treated with curcumin (10 μM) as previously described^43^, and then analyzed for myogenin expression. As shown in Fig. 5F, the level of myogenin mRNA was significantly decreased by curcumin treatment. In addition, the level of myogenin protein was also markedly reduced (Fig. 5G). Thus, p300 HAT activity is essential for myogenin gene expression.

### Impact of the Akt consensus motif on p300 function

**Figure 6:**
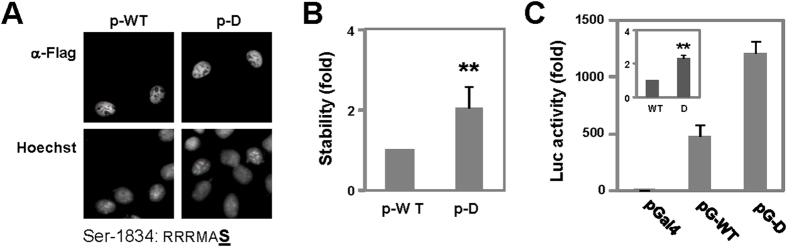
Role of Ser1834 in p300 stability and transcriptional activity. (**A**) HeLa cells were transfected with expression plasmids for Flag-tagged p300 wild-type (p-WT) and the Asp1834 mutant (p-D) and stained with an antibody against the Flag-tag for microscopy. The RRRMA**S** motif shown is the Akt consensus site. (**B**) Following ^35^S-methionine labeling and 8–12 hours of chase, equal amounts of whole cell extracts were immunopurified using a flag-tag antibody. Purified wild-type and mutant p300 were separated by SDS-PAGE and analyzed by PhosphorImager. Relative stability of the p300 mutant was presented as the fold change in relation to that of wild-type p300 (**p < 0.01, n = 4). (**C**) The cells were transfected with a Gal4 reporter, Gal4-DBD (pGal4) and Gal4 fusion of p300 wild-type (pG-WT) or the Asp1834 mutant (pG-D). Luciferase activity was normalized to β-Gal activities and presented as the fold change relative to the Gal4-DBD. Error bars are the standard deviations of triplicates. Inset is the transcriptional activity of Asp1834 mutant in fold change relative to that of wild-type Gal4-p300 (**p < 0.01, n = 3).

As Akt may regulate p300 function via phosphorylation of Ser1834 at the p300 C-terminus^21^,^23^, we thus wished to address if this consensus Akt site is important for control of p300 transcriptional activity. First, we generated a plasmid for mammalian expression of the full length p300 with the Ser1834 mutated to Asp which mimics the negative charge of O-phosphorylation. Immunoflorescence microscopy showed that the Asp1834 mutant was localized to the nucleus just as the wild-type p300 (Fig. 6A). A pulse-chase protocol demonstrated that metabolic stability of the p300 mutant increased by about 2-fold as compared to that of the wild-type p300 (Fig. 6B).

Next, we substituted the Ser1834 with Asp in the context of a Gal4-p300 fusion protein. Interestingly, the transcriptional activity of a Gal4 reporter was significantly enhanced by the Asp mutation (Fig. 6C). In addition, the activity of the reporter (Fig. 6C, inset) correlated consistently with the metabolic stability of the Asp1834 mutant (Fig. 6B). Thus, the Asp mutation may render the p300 more stable and thus more transcriptional active, suggesting that phosphorylation of the Ser1834 by Akt may be a molecular switch for the control of p300 function during myoblast differentiation.

## Discussion

We have investigated the molecular mechanism of p300 regulation during myogenic differentiation. Our data revealed that the levels of p300 protein are temporally maintained in RA-enhanced skeletal myocyte development. Most interestingly, this maintenance of p300 is observed post RA-enhanced lineage specification, which coincides with an augmentation of Akt phosphorylation. Furthermore, this regulation of p300 during the stages of myoblast differentiation appears to be mediated through Akt signaling and blunting of p300 inhibits myoblast differentiation. Thus, our data suggests a specific role for Akt in p300 regulation and myoblast differentiation. In addition, our study establishes the potential for exploiting p300 regulation and Akt activation as molecular pathways to decipher the complex signaling cascades involved in skeletal muscle development and to ultimately develop treatment strategies for muscle wasting disorders.

The transcriptional coactivator p300 is able to interact with over 200 different proteins and its function appears to be ubiquitously required by a myriad of cellular processes^8^. Nonetheless, depletion of p300 affects mouse development in a tissue specific manner^5^. In contrast to the solid evidence that exists for a p300 specific role in skeletal myogenesis, there is little information as to how p300 is regulated in this process. We found that treatment of pluripotent stem cells with RA at a concentration optimal for enhancing myogenic differentiation during EB formation, sustains the level of p300 protein for the late stages of myocyte development (Fig. 2). This maintenance of p300 by RA is particularly coupled with an increase in Akt phosphorylation (Figs 2 and 3). More importantly, knockdown of endogenous p300 protein or inhibits p300 HAT activity impairs myogenin expression and myoblast differentiation (Fig. 5). Given that p300 HAT activity is essential for skeletal myogenesis *in vivo* our findings suggest that RA enhances skeletal muscle development indirectly through Akt activation and p300 regulation.

Our studies also gained some molecular insights into the role of Akt in p300 regulation (Fig. 6). We have previously found that Akt positively regulates p300 function in proliferating cells^23^. Here, we show that the activation of Akt positively correlates with the levels of p300 protein and the efficacy of myogenic expression and myoblast differentiation (Fig. 4). Interpretation of the data obtained with an Akt isoform knockdown or overexpression approach may be confounded by the fact that Akt isoforms are highly homologous and able to compensate for the loss of each other. As a result, it is generally thought that the level of Akt expression rather than the specific activity of individual Akt isoforms determines the rate and extent of skeletal muscle development^44^.

The molecular mechanism underlying Akt specific signaling in skeletal muscle development is not well understood. While distinct tissue distribution of Akt isoforms may plays an important role^32^, determining the mechanisms by which external stimuli differentially activate Akt will provide biochemical insight into Akt regulation and the molecular basis for developing approaches to control specific Akt functionality. Another interesting question is to what extent a functional linkage between the specific activation of Akt and p300 regulation affects skeletal muscle development. Determining the impact of coactivator regulation on the differentiation and fusion of myoblasts will be important for advancing our knowledge regarding muscle regeneration and thus enable us to design phosphorylation-related tools and approaches to specifically exploit p300 activity or Akt activation in tissue engineering.

## Methods

### Cell culture and reagents

P19 pluripotent stem cells (ATCC) were maintained in the minimum essential medium α (Invitrogen) supplemented with 5% fetal bovine serum and 5% bovine calf serum at 37 °C and 5% CO_2_. After 4 days of EB formation in Petri dishes, the cells were then grown either in tissue culture dishes, or on coverslips coated with 0.1% gelatin for 5 days as previously described^35^. C2C12 myoblasts (ATCC) were maintained in Dulbecco’s Modified Eagle Medium (D-MEM) supplemented with 10% fetal bovine serum (HyClone) and differentiated in D-MEM supplemented with 2% horse serum as previously descirbed^43^. RA and DMSO were purchased from the Sigma-Aldrich and Akt inhibitor from the Calbiochem.

### Immunofluorescence microscopy

Cells were fixed on coverslips as described previously^45^ and incubated with antibody against muscle specific protein myosin heavy chain overnight at 4 °C, followed by the incubation with fluorescent secondary antibody (Molecular Probes) and Hoechst. Microscopic analysis was performed with a Zeiss Axiovert 200 M^46^. Images were captured with an AxioCam HR monochrome camera through fluorescence filters, and processed and merged by the Zeiss AxioVision Rel 4.6 software. For each coverslip of P19 myogenic conversion, about 100 fields of view were analyzed and the efficacies of differentiation were estimated based on the percentage of cells positively stained for myosin heavy chain in relation to the total cell populations as determined by nuclear Hoechst staining^36^. For each coverslip of C2C12 differentiation, about 5 images were analyzed and differentiation was defined as percentage of myocyte nuclei relative to the total number of nuclei. Antibody against myosin heavy chain was from MF20 hybridoma. Student *t*-tests were used for statistical analysis.

### Western and whole cell extracts

Cells were lysed by incubation in the whole cell extract buffer (10% glycerol, 50 mM Tris-HCl pH 7.6, 400 mM NaCl, 5 mM EDTA, 1 mM DTT, 1 mM PMSF, 1% NP-40) for 30 min at 4 °C for whole cell extraction as previously descrisbed^47^. Protein concentrations were determined by the Bradford Method (Bio-Rad). The proteins were separated by SDS-PAGE and transferred to the Immun-Blot PVDF membrane which was sequentially probed with the primary and secondary antibodies. Protein bands were quantified using Scion Image (Scion Corporation). Antibodies used were the following: p300 (SC-584, Santa Cruz Biotechnology), Akt (#9272, Cell Signaling), phosphorylated Akt (#9271, Cell Signaling), β-actin (A5441, Sigma-Aldrich), β-tubulin (E7 hybridoma), and myogenin (F5D hybridoma).

### Quantitative RT-PCR analysis

Total RNA was isolated using the Total RNA Kit I (Omega), and reverse-transcribed using a high-capacity cDNA Reverse Transcription Kit (Applied Biosystems). Quantitative PCR was performed using SYBR Green and ROX PCR Master Mix, and HotStarTaq DNA polymerase (Qiagen) as previously described^48^ with an Applied Biosystems 7500 Fast real-time PCR system. Quantification of the targets, normalized to the GAPDH endogenous reference and relative to calibrator control, was calculated using the formula 2^–ΔΔCT^.

### shRNA knockdown

C2C12 cells were grown in D-MEM supplemented with 10% fetal bovine serum to about 30% confluence and then infected with the p300 specific shRNA Lentiviral particles in the presence of Polybrene (5 μg/ml) according to the manufacturer’s protocol (Santa Cruz Biotechnology). A nonsilencing shRNA was used as a negative control. Puromycin (2 μg/ml) was used to select pooled stable clones as manufacturer suggested.

### Transfection and luciferase assays

Transfection of the plasmids was achieved by using ExGen 500 as described previously^23^. Plasmid for the p300 Asp1834 mutant was generated by using the parental wild-type p300 PCI/CMV plasmid^49^ and the site directed mutagenesis kit (Clontech). Plasmid for the Gal4-p300 fusion mutant was constructed using the Gal-p300 wild-type plasmid^50^ as the parental vector. The Gal-Reporter assays were carried out according to the manufacturer’s recommendation (Promega).

### Pulse-chase

HeLa cells were cultured for 16 hours after plating. The media was replaced with methionine-free media and the cells were pulsed for 2 hours with [^35^S]methionine (100 μCi/ml), and then chased for 8–12 hours in the regular medium as previously described^51^ The cells were then harvested for preparation of whole cell extracts. FLAG® antibody conjugates (Sigma-Aldrich) were used to immunoprecipitate the Flag-tagged p300 at 4 ^o^C for 2 hours. The immunopurified p300 was then separated by SDS-PAGE and analyzed using a PhosphoImager. For the immunoprecipitation, NaCl concentration of the whole cell extracts was adjusted to 150 mM and NP-40 to 0.1%.

## Additional Information

**How to cite this article**: Chen, J. *et al.* Molecular Basis for the Regulation of Transcriptional Coactivator p300 in Myogenic Differentiation. *Sci. Rep.* **5**, 13727; doi: 10.1038/srep13727 (2015).

## Supplementary Material

Supplementary Information
