# Supplementary material for: Molecular Basis for the Regulation of Transcriptional Coactivator p300 in Myogenic Differentiation
Source: Sci Rep. 2015 Sep 10;5:13727. doi: 10.1038/srep13727 (PMC4564756; doi:10.1038/srep13727)
Supplement: Supplementary Information [file srep13727-s1.pdf]

Supplementary Figure

Molecular Basis for the Regulation of Transcriptional Coactivator p300 in Myogenic Differentiation

Jihong Chen[2], Yinjian Wang[1], Munerah Hamed[1], Natascha Lacroix[1], and Qiao Li[1,2]

From the [1]Department of Cellular and Molecular Medicine, and the [2]Department of Pathology and Laboratory Medicine, Faculty of Medicine, University of Ottawa, Ottawa, Ontario, Canada

[*]Corresponding author: Q.L. (Qiao.Li@uOttawa.ca)

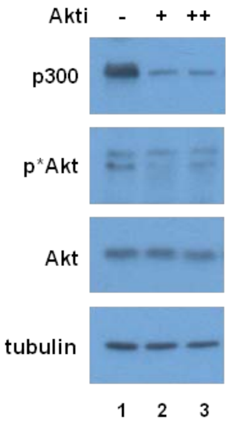

Figure S1. The full-length Western blots of the figure 4E.
The C2C12 cells were differentiated in the presence of Akt inhibitor IV (0.5, 1.0 μM). Levels of p300, Akt and phosphorylated Akt were analyzed by Western blotting on day 1 of differentiation. The blots were then stripped and reprobed for β-tubulin.
